# Tracking of TV and video gaming during childhood: Iowa Bone Development Study

**DOI:** 10.1186/1479-5868-8-100

**Published:** 2011-09-24

**Authors:** Shelby L Francis, Matthew J Stancel, Frances D Sernulka-George, Barbara Broffitt, Steven M Levy, Kathleen F Janz

**Affiliations:** 1 Department of Health and Human Physiology, University of Iowa, Iowa City, IA, USA; 2 Department of Epidemiology, University of Iowa, Iowa City, IA, USA; 3 Department of Preventive and Community Dentistry, University of Iowa, Iowa City, IA, USA

**Keywords:** physical activity, stability, sedentary behavior, adolescence

## Abstract

**Background:**

Tracking studies determine the stability and predictability of specific phenomena. This study examined tracking of TV viewing (TV) and video game use (VG) from middle childhood through early adolescence after adjusting for moderate and vigorous physical activity (MVPA), percentage of body fat (% BF), and maturity.

**Methods:**

TV viewing and VG use were measured at ages 5, 8, 11, and 13 (n = 434) via parental- and self-report. MVPA was measured using the Actigraph, % BF using dual-energy x-ray absorptiometry, and maturity via Mirwald predictive equations. Generalized Estimating Equations (GEE) were used to assess stability and logistic regression was used to predict children "at risk" for maintaining sedentary behaviors. Additional models examined tracking only in overfat children (boys ≥ 25% BF; girls ≥ 32% BF). Data were collected from 1998 to 2007 and analyzed in 2010.

**Results:**

The adjusted stability coefficients (GEE) for TV viewing were 0.35 (95% CI = 0.26, 0.44) for boys, 0.32 (0.23, 0.40) for girls, and 0.45 (0.27, 0.64) for overfat. For VG use, the adjusted stability coefficients were 0.14 (0.05, 0.24) for boys, 0.24 (0.10, 0.38) for girls, and 0.29 (0.08, 0.50) for overfat. The adjusted odds ratios (OR) for TV viewing were 3.2 (2.0, 5.2) for boys, 2.9 (1.9, 4.6) for girls, and 6.2 (2.2, 17.2) for overfat. For VG use, the OR were 1.8 (1.1, 3.1) for boys, 3.5 (2.1, 5.8) for girls, and 1.9 (0.6, 6.1) for overfat.

**Conclusions:**

TV viewing and VG use are moderately stable throughout childhood and predictive of later behavior. TV viewing appears to be more stable in younger children than VG use and more predictive of later behavior. Since habitual patterns of sedentarism in young children tend to continue to adolescence, early intervention strategies, particularly to reduce TV viewing, are warranted.

## Background

Childhood overweight and obesity rates have increased dramatically since 1990. The worldwide prevalence of childhood overweight and obesity increased from 4.2% in 1990 to 6.7% in 2010. In 2010, 43 million children were estimated to be overweight and obese, with another 92 million at risk of becoming overweight [1]. In the US, National Health and Nutrition Examination Survey (NHANES) data indicate that childhood obesity rates have tripled from 1980 to 2008 [2,3]. Previous studies have shown that increased sedentary behaviors, such as television viewing (TV), video game playing, computer game playing, and/or electronic game playing (VG), are linked to increased risk for overweight and obesity in the child population [4-7]. Based on this knowledge, public health officials have made reducing sedentary behaviors a focus for obesity prevention [8]. In order to implement successful prevention programs, a greater understanding of the age-related patterns of change, stability, and predictability of sedentary behaviors is needed.

Tracking studies quantify how well individuals maintain his/her rank within a cohort over time [9]. To do this, three main concepts must be addressed- the direction of change (whether the behavior increases or decreases), the stability of the behavior over time [9], and whether the behavior at an earlier time can be used to predict future behavior [9]. If sedentary behavior remains stable throughout childhood and into early adolescence, insight is provided as to when initial precursors and factors that determine this behavior occur and who should receive a targeted or high risk intervention early in life [10-12].

Many studies have focused on tracking of physical activity (PA) and inactivity [9,12-17], but fewer have assessed whether sedentary behaviors track in childhood or adolescence. Sedentary behaviors have been operationally defined as activities that consist of mostly sitting [18], and it has been suggested that this should be kept conceptually distinct from physical inactivity [19]. The latter, when commonly measured using objective monitors, such as accelerometers or heart rate monitors, reflects low movement counts or low heart rates. These measures are typically void of context, whereas sedentary behaviors are observable actions that children participate in within distinct situations (e.g., viewing TV). It is the actual sedentary behaviors, where the energy expenditure and movement intensity are assumed to be (relatively) low but the context of the activity is known, that are addressed in this study. A review by Biddle et al. examined the tracking of sedentary behaviors, and reported moderate-to-large coefficients for follow-up over several years, and smaller coefficients for longer time periods [18]. That review found evidence for slightly stronger tracking of TV viewing than other sedentary behaviors, but also noted that TV viewing may not be reflective of total sedentary time in children and adolescents as there appears to be a shift towards more VG use [18].

The aims of the current paper are three-fold: (1) to investigate the change in sedentary behaviors (specifically TV viewing and VG use) separately in boys and girls, (2) to examine the stability of these behaviors from middle childhood to early adolescence, and (3) to determine the predictability of future sedentary behaviors in childhood and early adolescence. Previous research has suggested that tracking of PA can be affected by gender, maturity, and level of adiposity; therefore, these factors, along with level of physical activity, were considered in this study of sedentary behaviors [10,20,21]. Stratification by gender and consideration of maturity, adiposity, and PA level reduced potential confounding and provided a longitudinal view of factors associated with the behaviors of interest (TV viewing and VG use).

## Methods

The current paper is a follow-up of a subsample of the Iowa Bone Development Study, a longitudinal study to improve understanding of bone health during childhood [22-24]. Study participants were recruited between 1998 and 2001 from a larger cohort of Midwestern children (n = 890) that were then participating in the Iowa Fluoride Study. The Iowa Fluoride Study population had been previously recruited (between 1992 and 1995) through eight Iowa hospitals immediately postpartum. The Iowa Bone Development Study participants are almost all (96%) white; nearly two-thirds of the participants' parents had some level of college education and a family income (at recruitment) of $20,000 per year or greater [22].

Sedentary behaviors, moderate and vigorous PA (MVPA), % BF, and maturity were gathered over an 8-year time period at four ages: 5, 8, 11 and 13 yr. A total of 434 children participated in measurements at age 5 (baseline) and at least one or more of the three follow-up measurements (ages 8, 11, and 13). The study was approved by The University of Iowa Institutional Review Board; written, informed consent was provided by the parents and assent by the children. Data were collected from 1998 to 2007 and were analyzed in 2010.

### Sedentary Behaviors

During each clinical visit, questionnaire data on TV viewing and VG use were collected. When the children were 5- and 8-years-old, parents were asked to report the average amount of time per day their child spent in these sedentary behaviors to the nearest quarter hour (i.e., On average, how many hours per day does your child spend watching any type of television including video movies? On average, how many hours per day does your child spend playing video games (such as Nintendo^®^) and/or computer games?). Parental reports are commonly used to assess these behaviors in young children [6,7,25] and have been shown to be moderately accurate when compared to direct observation (TV: r = 0.31 - 0.61; VG: r = 0.44 - 0.49) [26,27]. When the children were 11- and 13-years-old, self-report questionnaires were used with the following response categories: (1) < 1 hour · day^-1 ^or not at all; (2) ≥ 1 hour · day^-1 ^but < 2 hours · day^-1^; (3) ≥ 2 hours · day^-1 ^but < 3 hours · day^-1^; (4) ≥ 3 hours · day^-1 ^but < 4 hours · day^-1^; and (5) ≥ 4 hours · day^-1^. This method has been used in previous studies for children within this age range [28,29] (TV: r = 0.54) [30]. Amounts of time spent viewing TV and playing VG when the children were 5- and 8-years-old were categorized to match the response options when they were 11- and 13-years-old.

### MVPA

MVPA was assessed at each measurement period using Actigraph uniaxial physical activity monitors (model 7164). When compared to heart rate monitoring and indirect calorimetry, this method has been shown to be valid (r = 0.50 - 0.74) [31,32]. During a month in the autumn season, children aged 5 and 8 years were asked to wear the monitor during waking hours for 4 consecutive days (including one weekend day). When they were 11- and 13-years-old they were asked to wear the monitor during waking hours for 5 consecutive days (including two weekend days). Previous research has demonstrated less stable intraclass correlation coefficients in activity monitored PA in older children as compared to younger children, indicating the necessity for increased wear time for 11- and 13-year-olds [33]. To be considered as having complete PA data, children had to have worn the Actigraph monitor for at least 8 hours per day for a minimum of 3 days (within 15 months of the DXA scan). Children who had only 3 weekdays of data were not excluded from analysis. Movement count values were accumulated and summed over 1-minute intervals, as this was the shortest interval available at the time of measurement. MVPA minutes each day were used as a summary variable. The variable was derived using the cut point threshold of greater than 2999 movement counts per minute (ct · min^-1^) as defined by Treuth and collegues (R^2 ^= 0.84 and SEE = 1.36; calibrated against indirect calorimetry) [34].

### % Body Fat

Fat mass was determined using densiometry during clinical visits to the University of Iowa General Clinical Research Center by one of three qualified technicians. Specifically, whole-body scans using Hologic QDR 2000 dual energy x-ray absorptiometry (DXA) were conducted with software version 7.20B and fan-beam mode for 5- and 8-year-old children. The Hologic QDR 4500 DXA (Delphi upgrade) with software version 12.3 and fan-beam mode was used when they reached 11- and 13-years-of-age. Daily scans using the Hologic phantom were conducted to maintain quality-control.

To account for the differences between the two DXA machines, translation equations from QDR 2000 DXA measures to 4500 DXA measures were used for the data taken at 5 and 8 years of age. These equations were developed from a separate study developed specifically for comparing results with the two DXA machines. A total of 60 children (28 girls and 32 boys) 9.9 to 12.4 years of age (M = 11.4, SD = 0.4) were measured on both machines during one clinic visit in random order (TLB, unpublished observations, 2007). Total body fat mass (kilograms; kg) was derived from the scanned images. Percentage of body fat (% BF) was calculated based on total fat mass and body weight (total fat mass ÷ body weight × 100). The coefficient for determination (R^2^) for the QDR 2000 DXA data regressed onto the 4500 DXA was 0.9979. Actual observations were extremely tight around the regression line (TLB, unpublished data, 2007). % BF cut points (≥ 25% BF in boys and ≥ 32% BF in girls) were set to differentiate overfat children from healthy weight children in this study. Previous research has confirmed that these cut points are associated with significant increases in cardiovascular disease risk factors in children [35,36].

### Maturity

During each DXA visit, research nurses measured body mass (kilograms: kg) and height (centimeters; cm) using a Healthometer physician's scale (Continental, Bridgeview IL) and a Harpenden stadiometer (Holtain, United Kingdom). Both devices were calibrated routinely. Children were measured while wearing indoor clothes, without shoes. Sitting height was also measured when the children were 11 and 13 years. Maturity offset (year from peak height velocity) was calculated using predictive equations determined by Mirwald and colleagues [37]. Peak height velocity (or somatic maturity) was determined using height, weight, age, gender, sitting height, and leg length as predictors. These equations have been validated in white Canadian children and adolescents (R^2 ^= 0.91, 0.92, SEE = 0.49, 0.50). The maturity-offset variable was dichotomized as 0 (prior to peak height velocity, or pre-mature) or 1 (≥ peak height velocity, or mature).

### Statistical Analysis

Age-specific comparisons were conducted between boys and girls using the Student's t-test for age, height, weight, fat mass, % BF, and MVPA. The Cochran-Armitage trend test was used to determine if one sex reported significantly more TV viewing and/or VG use than the other sex. Bowker's test of symmetry was used to evaluate possible directionality of movement between categories of TV viewing and VG use. Stability of TV viewing and VG use over time was assessed with weighted kappa coefficients, Kendall Tau b correlations, and Generalized Estimating Equations (GEE). Weighted kappa coefficients provide a method of quantifying the stability of the TV viewing and VG use measures, while Kendall Tau b correlations measure the association between TV viewing and VG use measures from one measurement year to the next. GEE provides a method of analyzing correlated data in which subjects are assessed at different points in time and have a varying number of data points. The GEE models were adjusted for maturity, overfat (at age 5 and concurrently, i.e., current measurement age), and MVPA (at age 5 and concurrently) in boys and girls separately. GEE was also used for a subset of overfat children (n = 34, boys and girls combined to maintain power) to examine if tracking of TV and VG is greater in children who are already overfat, i.e., already at risk. The overfat model was adjusted for maturity, sex, and MVPA (at age 5 and concurrently). Logistic regression was used to determine the odds of remaining in the upper category of TV viewing and VG use at ages 8, 11, and 13 based on being in the upper category at age 5, relative to children in the lower categories at age 5. The data for each analysis were divided into quintiles, with the top quintile being used as the upper category. The upper category for both boys' and girls' TV viewing was > 3 hours · day^-1^. The upper categories for boys' VG use were > 1 hour · day^-1 ^at age 5, > 2 hours · day^-1 ^at ages 8 and 11, and > 3 hours · day^-1 ^at age 13. The upper category for girls' VG use was > 1 hour · day^-1 ^except age 13, where the upper category was > 2 hours · day^-1^. This model also accounted for maturity, overfat (age 5 and concurrently), and MVPA (age 5 and concurrently). A secondary analysis using only the overfat children was also examined. This model also accounted for maturity, sex, and MVPA (at age 5 and concurrently). All statistical analyses were conducted using SAS version 9.1.3. and were analyzed separately by gender (with the exception of the overfat children analyses, where boys and girls were combined). Results with p < 0.05 were considered statistically significant.

## Results

### Characteristics of Participants

**Figure 1:**
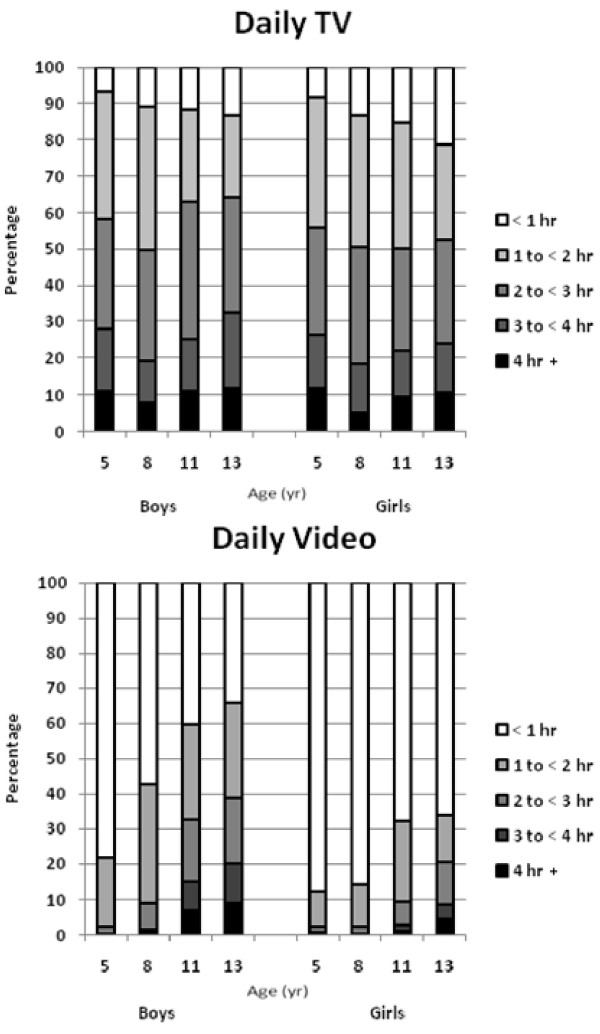
**Percentages of children in TV and VG categories at each age**. Boys (age 5: n = 205; age 8: n = 193; age 11: n = 171; age 13: n = 168). Girls (age 5: n = 229; age 8: n = 222; age 11: n = 211; age 13: n = 189).

The characteristics of the participants at the time of each measurement (ages 5, 8, 11, and 13 yr), including age, height, weight, fat mass, % BF, and MVPA, are provided in Table 1. At all ages, boys were more active than girls (p < 0.05). Time in MVPA increased from age 5 to 11 for boys, and then decreased at age 13. Girls' time in MVPA increased from age 5 to 8, and then decreased at ages 11 and 13. The proportion of children in each category for TV viewing and VG use at each measurement age are provided in Figure 1. More than half of the entire sample reported watching more than 2 hours of TV per day at each measurement age. Boys spent more time playing VG than girls at all ages (p < 0.05), and the time spent playing VG increased for both boys and girls over the four measurement periods.

**Table 1:** Participant Characteristics

	Age 5		Age 8		Age 11		Age 13	
	
	Boys	Girls	Boys	Girls	Boys	Girls	Boys	Girls
	
	(n = 205)	(n = 229)	(n = 193)	(n = 222)	(n = 171)	(n = 211)	(n = 168)	(n = 189)
Age (yr)	5.2 ± 0.4	5.3 ± 0.5*	8.7 ± 0.6	8.7 ± 0.6	11.2 ± 0.3	11.2 ± 0.3	13.3 ± 0.4	13.3 ± 0.4

Height (cm)	112 ± 6	111 ± 6	134 ± 7*	132 ± 7	149 ± 8	149 ± 8	163 ± 10*	160 ± 7

Weight (kg)	21 ± 4	20 ± 4	33 ± 10	32 ± 9	45 ± 13	44 ± 12	58 ± 16	56 ± 15

Fat Mass (kg)	3.9 ± 2.1	4.6 ± 2.4*	8.0 ± 6.3	9.2 ± 5.9	12.7 ± 9.2	14.2 ± 8.7*	15.4 ± 11.6	17.9 ± 10.6*

% BF^a^	18.8 ± 5.3	22.8 ± 6.0*	22.6 ± 9.3	27.7 ± 8.8*	26.4 ± 10.9	30.5 ± 9.9*	23.4 ± 12.6	29.3 ± 9.9*

MVPA (minutes · day^-1^)^b^	31.5 ± 15.9*	24.5 ± 13.1^c^	38.6 ± 21.2*	25.9 ± 15.5^d^	42.0 ± 22.1*	22.7 ± 13.9	31.6 ± 18.1*	19.5 ± 13.1^e^

TV (≥ 2 hr · day^-1^)^f ^(%)	58	56	50	51	63	50	64^†^	52

VG (≥ 1 hr · day^-1^)^f ^(%)	22^†^	12	42^†^	14	60^†^	32	66^†^	34

Values are presented as mean ± SD.

SD, standard deviation.

^a ^Percentage of body fat determined by DXA scan.

^b ^moderate-to-vigorous physical activity.

^c ^n = 228.

^d ^n = 221

^e ^n = 187.

Response categories: (1) < 1 hr · day-1 or not at all; (2) ≥ 1 hr · day-1 but < 2 hr · day-1; (3) ≥ 2 hr · day-1 but < 3 hr · day-1;

(4) ≥ 3 hr · day-1 but < 4 hr · day-1; and (5) ≥ 4 hr · day-1.

* Age-specific comparison of boys and girls using Student's t-test (p < 0.05).

^† ^Age-specific comparison of boys and girls using the Cochran-Armitage trend test (p < 0.05).

There was an increase in TV viewing for boys from age 8 to 13 (p < 0.05) (Table 2). Boys' VG use increased significantly at each age (p < 0.005), except from age 11 to 13, when there was no significant increase. Girls' TV viewing decreased from age 5 to 8 (p < 0.05), but then leveled off. VG use for girls showed no significant increase from age 5 to 8, but did increase significantly thereafter (p < 0.05).

**Table 2:** Bowker's test of symmetry for percentage of change in daily TV and VG time between ages 5, 8, 11, and 13

		Age 5-8	Age 5-11	Age 5-13	Age 8-11	Age 8-13	Age 11-13
**TV Viewing**							

Boys	Increase	23	36	36	43*	43*	34

	Decrease	41	38	37	28	24	32

							

Girls	Increase	22	27	32	35	37	35

	Decrease	41*	39	41	29	33	38

							

**Video Gaming**							

Boys	Increase	33**	54***	61***	45***	53***	39

	Decrease	10	8	8	15	13	35

							

Girls	Increase	9	27**	31***	26**	32***	28*

	Decrease	7	8	7	5	9	17

* p < 0.05; ** p < 0.01; *** p < 0.001.

Boys (age 5: n = 205; age 8: n = 193; age 11: n = 171; age 13: n = 168.)

Girls (age 5: n = 229; age 8: n = 222; age 11: n = 211; age 13: n = 189.)

Weighted kappa coefficients (Table 3) for boys' TV viewing showed slight (0.12 to 0.19) to fair (0.22 to 0.29) agreement. Their VG use coefficients showed only slight (0.01 to 0.14) agreement. The weighted kappa coefficients for girls' TV viewing time showed slight (0.09 to 0.20) to fair (0.21 to 0.34) agreement. Similarly, their VG use coefficients also showed slight (0.01 to 0.18) to fair (0.22 to 0.34) agreement. Landis and Koch characterized coefficients ranging from 0 to 0.20 as slight agreement, and coefficients ranging from 0.21 to 0.40 as fair agreement [38]. Kendall Tau b correlation coefficients are shown in Table 4. Boys' TV coefficients ranged from 0.20 to 0.40. Their VG use coefficients ranged from 0.04 to 0.18. Girls' TV coefficients ranged from 0.09 to 0.44. Their VG use coefficients ranged from 0.03 to 0.35.

**Table 3:** Weighted kappa coefficients for stability of daily TV and VG time between ages 5, 8, 11, and 13

	Age 5-8	Age 5-11	Age 5-13	Age 8-11	Age 8-13	Age 11-13
	**(n = 193 boys, 222 girls)**	**(n = 171 boys, 211 girls)**	**(n = 168 boys, 189 girls)**	**(n = 161 boys, 205 girls)**	**(n = 158 boys, 185 girls)**	**(n = 152 boys, 180 girls)**

**TV Viewing**						

Boys	0.29** (0.20, 0.38)	0.12* (0.02, 0.22)	0.17* (0.07, 0.27)	0.19* (0.09, 0.28)	0.22** (0.12, 0.32)	0.24** (0.13, 0.36)

Girls	0.34** (0.25, 0.42)	0.21** (0.11, 0.30)	0.09* (-0.01, 0.19)	0.30** (0.22, 0.39)	0.20* (0.11, 0.30)	0.17* (0.07, 0.26)

						

**Video Gaming**						

Boys	0.14* (0.02, 0.26)	0.05* (-0.01, 0.10)	0.01* (-0.04, 0.07)	0.10* (0.01, 0.19)	0.11* (0.04, 0.19)	0.13* (0.02, 0.25)

Girls	0.34** (0.17, 0.51)	0.09* (0.00, 0.17)	0.08* (-0.01, 0.18)	0.22** (0.11, 0.32)	0.02* (-0.05, 0.10)	0.18* (0.08, 0.29)

Presented as kappa (95% confidence interval)

Landis and Koch (1977) proposed classifications for the interpretation of a weighted kappa value. * slight; ** fair.

**Table 4:** Kendall Tau b correlation coefficients for stability of daily TV and VG time between ages 5, 8, 11, and 13

	Age 5-8	Age 5-11	Age 5-13	Age 8-11	Age 8-13	Age 11-13
	Boys: n = 193	Boys: n = 171	Boys: n = 168	Boys: n = 161	Boys: n = 158	Boys: n = 152
	Girls: n = 222	Girls: n = 211	Girls: n = 189	Girls: n = 205	Girls: n = 185	Girls: n = 180
**TV Viewing**						
Boys	0.40***	0.20**	0.24***	0.28***	0.30***	0.29***
Girls	0.44***	0.27***	0.09	0.41***	0.28***	0.25***
						
**Video Gaming**						
Boys	0.16*	0.15*	0.04	0.13	0.18**	0.15*
Girls	0.35***	0.15*	0.12	0.32***	0.03	0.27***

* p < 0.05; ** p < 0.01; *** p < 0.001

GEE analyses for TV viewing and VG use for boys, girls, and overfat children (boys and girls combined) are summarized in Table 5. After adjustment, the boys' and girls' coefficients remained virtually unchanged, indicating that maturity, overfat (age 5 and concurrent), and MVPA were not confounding the results. The overfat children's coefficients were altered slightly after adjustment; being female was the only significant variable in the adjusted model for TV viewing (p < 0.05). None of the variables were significant in the overfat children's VG use model.

**Table 5:** Generalized estimating equation coefficients and odds (predictability) of TV and VG for boys, girls, and those classified as overfat (n = 205 boys, 229 girls, 34 overfat)

	Unadjusted	Adjusted	Unadjusted	Adjusted
	
	Stability Coefficient	Stability Coefficient	Odds Ratio	Odds Ratio
**TV Viewing**				
Boys	0.35 (0.26, 0.44)	0.35 (0.26, 0.44)^a^	3.0 (1.9, 4.8)	3.2 (2.0, 5.2)^a^
Girls	0.34 (0.26, 0.43)	0.32 (0.23, 0.40)^a^	3.2 (2.1, 4.9)	2.9 (1.9, 4.6)^a^
Overfat^b^	0.41 (0.23, 0.59)	0.45 (0.27, 0.64)^c^	3.7 (1.5, 9.0)	6.2 (2.2, 17.2)^c^
				
**Video Gaming**				
Boys	0.15 (0.05, 0.25)	0.14 (0.05, 0.24)^a^	1.9 (1.1, 3.2)	1.8 (1.1, 3.1)^a^
Girls	0.24 (0.09, 0.39)	0.24 (0.10, 0.38)^a^	3.4 (2.1, 5.8)	3.5 (2.1, 5.8)^a^
Overfat^b^	0.37 (0.16, 0.57)	0.29 (0.08, 0.50)^c^	2.7 (0.7, 10.6)	1.9 (0.6, 6.1)^c^

Values are presented as point estimate (95% confidence interval).

^a ^Adjusted for maturity, overfat (age 5 and concurrent), and MVPA (age 5 and concurrent).

b Cut-points for being overfat: Boys ≥ 25% BF, Girls ≥ 32% BF.

^c ^Adjusted for maturity, sex, and MVPA (age 5 and concurrent).

Odds of being in upper category at subsequent ages for for children in upper category at age 5, relative to children in lower categories at age 5. The upper category for both boys' and girls' TV was > 3 hours · day^-1.^

The upper categories for boys' VG were > 1 hour · day^-1 ^at age 5, > 2 hours · day^-1 ^at ages 8 and 11, and > 3 hours · day^-1 ^at age 13. The upper category for girls' video was > 1 hour · day^-1 ^except age 13, where the upper category was > 2 hours · day^-1^.

Logistic regression was used to determine if we could predict high levels of TV viewing or VG use later in life (age 13) based on age 5 levels (Table 5). Both the unadjusted and adjusted OR, as an estimate of relative risk (RR), for boys' and girls' TV viewing were approximately 3.0. The crude OR for TV viewing for the overfat children was 3.7 (95% CI = 1.5, 9.0); after adjustment it was 6.2 (95% CI = 2.2, 17.2). Gender (specifically being female) was significant in this adjusted model (p < 0.05). The crude OR for VG use for the overfat children was 2.7 (95% CI = 0.7, 10.6) and 1.9 (95% CI = 0.6, 6.1) after adjustment; none of the variables were significant in this model.

## Discussion

Increased sedentary behaviors are linked to increased risk for overweight and obesity in the child population [4-7]. This study examined the tracking of select sedentary behaviors (TV viewing and VG use) in one group of children from approximately age 5 to age 13. We report increased sedentary behavior (especially VG use) over time, slight to fair stability of TV viewing and VG use over time, and the tendency of early values (especially TV viewing) to predict later values. Additionally, overfat 5-year-old girls who watched a great deal of TV were highly likely to continue this behavior (TV viewing) as they aged.

### TV and VG

The amount of time spent watching TV stayed relatively stable over time, with more than half of the sample reporting that they watched more than two hours of TV daily at every measurement age. The current recommendation by the American Academy of Pediatrics is that children should limit their total media time to 1 to 2 hours per day [39]. The children in our sample were exceeding that recommendation with TV viewing alone. The odds of remaining in the "at risk" (highly sedentary) group are higher for TV viewing than for VG use. It has been suggested by Sturm [40] that this might be due to secular trends and the length of time that TV has been available compared to newer forms of media (i.e., computer and VG games). TV viewing has become a prevalent sedentary behavior in present-day society and it has been identified that prolonged TV viewing may be associated with weight gain. This weight gain could be caused by a reduction in energy expenditure if kids are watching TV instead of participating in active play or sport and/or by increasing caloric intake by snacking while viewing or altering eating patterns based on food advertising [40]. Even though VG use was found to be much less stable than TV viewing, VG time increased roughly three-fold for both boys and girls. These results might be explained in two ways: 1) that VG use does increase as children age from 5 to 13, and/or, 2) that VG use is gaining popularity as a secular trend at all ages due to targeting and availability of this technology to younger and younger children [40]. Additional research on VG usage in children is needed to determine if either explanation is plausible.

### Maturity, Overfat, and MVPA

Surprisingly, neither the TV viewing nor VG use GEE and OR results were altered significantly after adjustment for maturity, overfat, or MVPA, indicating that these potential confounders do not substantially affect TV viewing or VG participation. The results for TV viewing remained relatively stable for both boys and girls. However, girls' VG use was more stable than boys', even though more boys reported playing VG. This suggests that, even though a large number of boys (66% reported ≥ 1 hr · day^-1 ^at age 13) participate in VG use, the girls who play at a young age continue to play throughout childhood and into adolescence. In fact, the girls in the present study are over 3 times as likely to remain in the "at risk" category for VG if they were in this category at age 5. Unfortunately, data were not collected that could explain this gender difference, but this does suggest that for boys a broad, population-based intervention approach would be warranted since "at risk" status would be expected to change, while the girls reporting VG use at a young age, or those in the "at risk" group, are likely to remain so and therefore a specific, targeted intervention would be warranted. Regardless, our findings are cause for concern due to the increasing availability of VG which is marketed toward younger populations. In addition, we suspect that, as VG is marketed more toward young girls, there will also be an increase in the proportion of girls being classified in the "at risk" category.

Similar to previous research, we found that boys were more active (MVPA) than girls at each measurement point [10,13,15]. However, boys also watched more TV at age 13 than girls and played more VG than girls at every age, suggesting that PA and sedentary behaviors are independent. This is consistent with research conducted by Biddle et al. [18] which suggested that TV viewing and VG use were largely uncorrelated with PA in adolescents, indicating that there is time for an individual to be both active and sedentary. Our results contribute to the literature, suggesting that being both physically active and sedentary are distinct behaviors and should be adjusted for when conducting research.

Additionally, our results coincide with previous knowledge that MVPA decreases as children age [41]. Unfortunately, TV viewing and VG use do not appear to be decreasing with maturity in the same manner. Decreasing levels of MVPA combined with consistent or increasing amounts of TV viewing and VG use as children age may lead to future health problems.

### Overfat Girls and TV

The subgroup of overfat children analyzed were six times as likely to remain in the upper category for TV viewing at later ages if they were in the upper category at age 5 (from adjusted analyses). Gender (being female) was the only significant co-variate in this model, suggesting that overfat girls are likely to begin watching TV at a young age and continue watching as they age. This "at risk" group may benefit from targeted interventions. However, due to the small sample size of overfat girls in our study, more research is needed to determine if TV viewing time indeed tracks better in the overfat, female population.

Limitations of our study include limited representation of minorities and children from low socioeconomic status (SES) households. Also, parental report of children's sedentary behavior is less accurate than direct observation [42]. However, this study is one of the few to investigate the longitudinal trends of sedentary behavior in a relatively large sample of children. Additional study strengths include the use of objective measures of % BF (DXA) and physical activity (Actigraph). Finally, our ability to examine sedentary behaviors from middle childhood through adolescence enhances our understanding of the pattern of change, stability, and predictability of these behaviors.

## Conclusions

With the exception of overfat girls, the tracking of TV viewing and VG use was at best moderate, suggesting that some children who initially participate in extremely high or relatively low levels of sedentary behavior may shift into other categories over time. Our results are consistent with those found in the review previously mentioned by Biddle et al., that tracking coefficients for shorter time periods are larger than coefficients for larger time periods [18]. Our results also indicate that overfat girls maintain stable sedentary behavior patterns over time which suggests the need for "high-risk," targeted interventions aimed at preventing excessive sedentary behavior patterns early in life.

## Competing interests

The authors declare that they have no competing interests.

## Authors' contributions

SF participated in the drafting of the manuscript. KJ participated in the design and coordination of the study, and helped to draft the manuscript. MS participated in the drafting of the manuscript. DSG participated in the drafting of the manuscript. BB performed the statistical analysis. SL participated in the design and coordination of the study. All authors read and approved the final manuscript.
